# HER3 as biomarker and therapeutic target in pancreatic cancer: new insights in pertuzumab therapy in preclinical models

**DOI:** 10.18632/oncotarget.2231

**Published:** 2014-07-17

**Authors:** GaëLle Thomas, Thierry ChardèS, NadèGe Gaborit, Caroline Mollevi, Wilhem Leconet, Bruno Robert, Nina Radosevic-Robin, FréDéRique Penault-Llorca, CéLine Gongora, Pierre-Emmanuel Colombo, Yassamine Lazrek, Rui Bras-Goncalves, Ariel Savina, David Azria, Hervé Bazin, André PèLegrin, Christel Larbouret

**Affiliations:** ^1^ IRCM, Institut de Recherche en Cancérologie de Montpellier, Montpellier, F-34298, France; INSERM, Unit 896, Montpellier, F-34298, France; Université Montpellier1, Montpellier, F-34298, France; ICM, Montpellier, France; ^2^ Institut Roche de Recherche et Médecine Translationnelle, Boulogne Bilancourt, France; ^3^ Department of Biopathology, The Jean Perrin Comprehensive Cancer Center and ERTICa Research Group, University of Auvergne EA4677, Clermont-Ferrand, France; ^4^ Millegen SA, F-31681, Labège, France; ^5^ Unité de Biostatistiques, ICM Val d'Aurelle, Montpellier, France; ^6^ Roche SAS Scientific Partnerships, Boulogne Billancourt, France; ^7^ CisBio Bioassays, Le Codolet, France

**Keywords:** HER3, HER2, pertuzumab, pancreatic cancer

## Abstract

The anti-HER2 antibody pertuzumab inhibits HER2 dimerization and affects HER2/HER3 dimer formation and signaling. As HER3 and its ligand neuregulin are implicated in pancreatic tumorigenesis, we investigated whether HER3 expression could be a predictive biomarker of pertuzumab efficacy in HER2^low^-expressing pancreatic cancer. We correlated *in vitro* and *in vivo* HER3 expression and neuregulin dependency with the inhibitory effect of pertuzumab on cell viability and tumor progression. HER3 knockdown in BxPC-3 cells led to resistance to pertuzumab therapy. Pertuzumab treatment of HER3-expressing pancreatic cancer cells increased HER3 at the cell membrane, whereas the anti-HER3 monoclonal antibody 9F7-F11 down-regulated it. Both antibodies blocked HER3 and AKT phosphorylation and inhibited HER2/HER3 heterodimerization but affected differently HER2 and HER3 homodimers. The pertuzumab/9F7-F11 combination enhanced tumor inhibition and the median survival time in mice xenografted with HER3-expressing pancreatic cancer cells. Finally, HER2 and HER3 were co-expressed in 11% and HER3 alone in 27% of the 45 pancreatic ductal adenocarcinomas analyzed by immunohistochemistry. HER3 is essential for pertuzumab efficacy in HER2^low^-expressing pancreatic cancer and HER3 expression might be a predictive biomarker of pertuzumab efficacy in such cancers. Further studies in clinical samples are required to confirm these findings and the interest of combining anti-HER2 and anti-HER3 therapeutic antibodies.

## INTRODUCTION

Pancreatic cancer remains one of the most aggressive tumors. As at diagnosis 60 to 80% of patients already have locally advanced or metastatic disease [1], only palliative therapy is possible and the 5-year survival rate is lower than 5% [2]. In an effort to find new treatments, the molecular mechanisms involved in pancreatic cancer development are currently investigated and EGFR family members [3] have emerged as relevant and promising therapeutic targets. The significant, but modest, increase in median survival obtained with the erlotinib/gemcitabine combination illustrates the potential and the challenges of anti-EGFR therapies in pancreatic cancer [4]. Moreover, a better selection of the patients who could benefit from these novel therapies could improve their efficacy and the prognosis, as demonstrated for the anti-HER2 antibody trastuzumab in breast cancers. Indeed, trastuzumab blocks the growth of breast tumors that strongly express HER2 or with *HER2* gene amplification, but is not efficient in cancers with normal or low HER2 protein level. It is critical to emphasize that trastuzumab therapeutic activity could have been underestimated by testing it in patients who had not been selected based on their HER2 status [5].

We have recently demonstrated the interest of targeting EGFR/HER2 heterodimers in HER2^low^- expressing pancreatic cancer [6]. The combination of cetuximab and trastuzumab had a synergistic anti-tumor effect that was mainly due to the modification of the distribution of homo- and hetero-dimers, with disruption of EGFR/HER2 dimers and increased homodimer formation. To date the only HER2-targeting drug known to disturb ligand-activated HER2 dimerization is pertuzumab. This antibody binds to a different HER2 epitope than trastuzumab and thereby has a therapeutic effect in preclinical studies that does not strictly require HER2 protein overexpression [8]. Nevertheless, pertuzumab was recently approved, in combination with trastuzumab and docetaxel, only for the treatment of HER2-positive (HER2^high^) metastatic breast cancer [9]. Moreover, pertuzumab inhibits neuregulin-stimulated cell growth mediated by HER2/HER3 dimers in breast cancer, whereas trastuzumab is more efficient in the absence of neuregulin [8]. These two antibodies seem to regulate differently HER2 homo- and hetero-dimers and to have different ligand dependencies.

HER3 is the main HER2 partner involved in pertuzumab response in ovarian cancer [10], but it could also influence pancreatic cancer tumorigenesis [11] because it is often overexpressed in pancreatic cancer and HER3 high expression correlates with advanced disease stages and lower overall survival [12, 13]. Although HER3 has a very weak intracellular tyrosine kinase activity, its neuregulin-triggered transactivation by other members of the EGFR family induces direct phosphorylation of the six binding sites for the p85 regulatory subunit of PI3K, resulting in activation of the AKT signaling cascade [14]. Furthermore, HER3 is a key player in tumor cell resistance to EGFR- and HER2-targeted therapies, particularly in pancreatic cancer where it regulates EGFR signaling by modulating the response to erlotinib or cetuximab [13]. HER3-mediated AKT signaling participates also in gemcitabine resistance [15]. In HER2-amplified breast tumors, resistance to trastuzumab or to tyrosine kinase inhibitors has been associated with HER3 expression [15].

In this study, we investigated whether HER3 expression could affect the therapeutic response to pertuzumab in HER2^low^ pancreatic cancer by analyzing *in vitro* and *in vivo* pertuzumab effects in HER2^low^ pancreatic cancer cell lines that express or not HER3. We show that the inhibitory effect of pertuzumab on cell viability and tumor progression in pancreatic cancer xenografts correlates with HER3 protein expression and is neuregulin-dependent. Accordingly, HER3 knockdown led to resistance to pertuzumab therapy. We also found that HER3 mRNA level and cell surface expression were increased after pertuzumab treatment. Based on these results we tested the effects of combining pertuzumab with the anti-HER3 therapeutic antibody 9F7-F11 [17]. The combined treatment enhanced tumor growth inhibition in pancreatic cancer xenografts, suggesting that it might represent a new potential therapy for pancreatic cancers that co-express HER2 and HER3. Finally, we analyzed the co-expression of HER2 and HER3 by immunohistochemistry (IHC) in 45 pancreatic ductal adenocarcinomas (PDAC).

## RESULTS

### HER3 expression and NRG1β1-induced proliferation in pancreatic cancer cells

**Figure 1:**
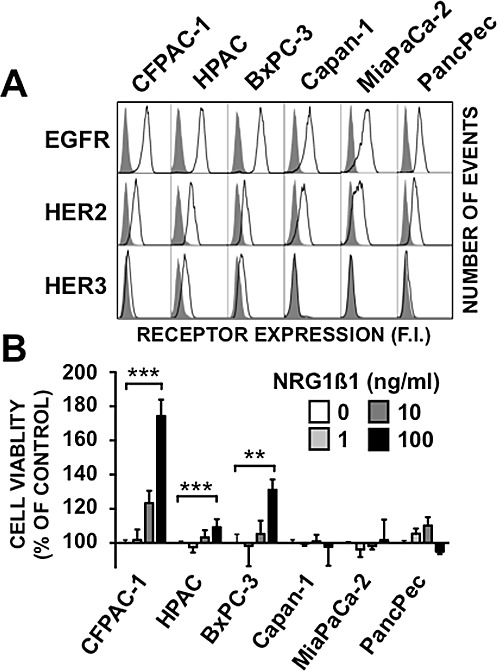
Characteristics of the six pancreatic cancer cell lines A, Flow cytometry analysis of EGFR, HER2 and HER3 expression in the six pancreatic cancer cell lines. Black lines depict cell surface staining with the anti-EGFR, anti-HER2 or anti-HER3 antibodies. The filled dark grey peaks represent controls, obtained with cells incubated only with the FITC-labeled secondary antibody. B, The six cell lines were serum-starved for 24h and then incubated with 1, 10 or 100 ng/ml of NRG1β1 for 5 days. Cell proliferation was measured by MTS. Data are the mean ± SD, n=4. *** p < 0.001, ** p < 0.01.

The expression of HER family members and NRG1β1-induced proliferation was assessed in five pancreatic cancer cell lines that harbor *KRAS* mutations and in one cell line (BxPC-3) wild type. All of them were wild type PTEN/PIK3CA. These data are consistent with patterns described in pancreatic tumors (supplementary Table 1). Flow cytometry showed that HER3 was expressed in CFPAC-1, HPAC and BxPC-3, but not in Capan-1, MiaPaCa-2 and PancPec cells. Comparably moderate HER2 and higher EGFR levels were observed in all cell lines (Figure1A). HER4 expression could not be detected in the six cell lines [data not shown]. Incubation with NRG1β1 induced cell proliferation in a dose-dependent manner only in the HER3-positive (BxPC-3, CFPAC-1 and HPAC) cell lines (Figure 1B). However, the finding that NRG1β1 effect was highest in CFPAC-1 cells, which had the lowest HER3 expression level, indicates that it is independent of the HER3 membrane expression level.

### Pertuzumab inhibits growth of HER3-positive pancreatic tumors both *in vitro* and *in vivo*

**Figure 2:**
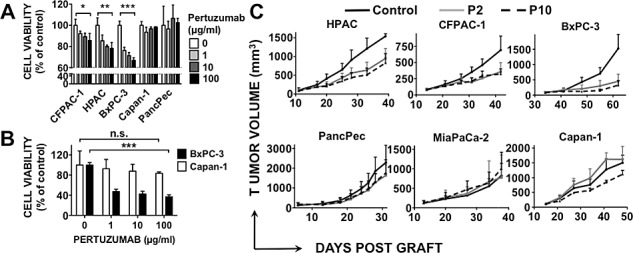
Effect of pertuzumab on HER3-positive or -negative pancreatic cancer cell lines Cells were directly incubated with pertuzumab for 5 days (A) or serum-deprived for 24h and stimulated with 10 ng/ml NRG1β1 added two hours after pertuzumab treatment (B). Cell proliferation was analyzed by MTS. Data are the mean ± SD, n=3. Results were expressed relative to untreated cells. *** p < 0.001, ** p < 0.01 * p < 0.05, n.s., not significant. C, Pancreatic cancer cells were xenografted in nude mice that were then treated with 2 or 10 mg/kg pertuzumab (P2 and P10) or sterile PBS twice/week. Results are presented as the mean tumor volume of each group. Bars = SEM.

We then investigated the ability of pertuzumab to inhibit the growth of the six pancreatic cancer cell lines after stimulation, or not, by NRG1β1. In the absence of ligand stimulation, pertuzumab reduced the growth of the HER3-positive cell lines in a dose-dependent manner [between 10% and 35%], but did not affect the viability of the HER3-negative cell lines (Figure 2A). Pertuzumab growth inhibition of the HER3-positive pancreatic cancer cell line BxPC-3 was significantly increased following stimulation with NRG1β1 (60% inhibition with neuregulin stimulation versus 35% without it) (Figure 2B). Conversely, no NRG1β1 effect was observed in HER3-negative Capan-1 cells (Figure 2B). These results demonstrate that pertuzumab is efficient in HER3-positive pancreatic cancer cells independently of NRG1β1 stimulation; however, neuregulin signaling through HER3 potentiates its inhibitory effect on pancreatic cell growth.

We evaluated the effect of pertuzumab on growth of tumors obtained by xenografting the six pancreatic cancer cell lines. The volume of HER3-positive tumor xenografts was significantly smaller in mice treated with 2 or 10 mg/kg pertuzumab than in animals treated with vehicle alone (controls) (p=0.0052 for HPAC; p=0.0269 for CFPAC-1; p<0.001 for BxPC-3 cells) (Figure 2C). No significant difference was observed between the 2 and 10 mg/kg regimens in the three models, suggesting that 2 mg/kg is sufficient to saturate the low HER2 receptor expression. In contrast, pertuzumab had almost no effect on growth of tumors derived from HER3-negative pancreatic cancer cells, demonstrating that pertuzumab is efficient *in vivo* only in HER3-positive pancreatic tumor xenografts.

### HER3 knockdown abrogates pertuzumab efficacy in BxPC-3 pancreatic cancer cells *in vitro* and *in vivo*

**Figure 3:**
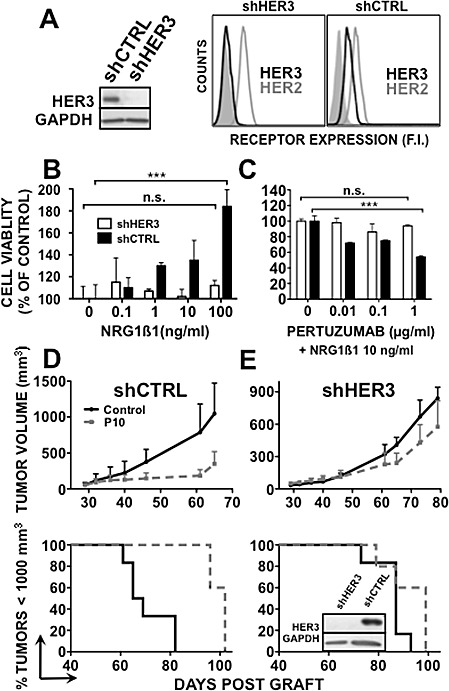
*HER3* knockdown abrogates pertuzumab efficacy in BxPC-3 cells HER3 expression was assessed by western blotting or cytometry [A] in BxPC-3 shHER3 and shCTRL cells. B, shHER3 and shCTRL BxPC-3 cells were serum-starved for 24h and then incubated with NRG1β1 for 5 days (left panel). C, ShHER3 and shCTRL BxPC-3 cells were serum-starved for 24h and then incubated with NRG1β1 and pertuzumab for 5 days (right panel). Cell proliferation was analyzed by MTS. Data are the mean ± SD. Results are expressed as percentage of growth relative to control (untreated cells). *** p < 0.001; n.s., not significant. ShCTRL (D) and shHER3 (E) BxPC-3 cells were xenografted in nude mice that were then treated with 10 mg/kg pertuzumab or sterile PBS twice per week. Results are presented as the mean tumor volume of each group during the treatment and Kaplan-Meier survival curves. Bars = SEM.

To further investigate HER3 implication in the response to pertuzumab, we generated a BxPC-3 cell line in which HER3 expression was abrogated by stable knockdown of *HER3* by shRNA (shHER3 BxPC-3). BxPC-3 cells that stably express the pSIREN-shLuc vector alone were used as negative control (shCTRL BxPC-3). HER2 expression was not affected and was similar in both cell lines (Figure 3A). The dose-dependent positive effect of NRG1β1 on cell proliferation observed in parental BxPC-3 cells was inhibited in shHER3 BxPC-3 cells, but not in shCTRL BxPC-3 cells (Figure 3B). Treatment with 1 μg/ml pertuzumab significantly inhibited proliferation of NRG1β1-stimulated shCTRL BxPC-3 cells and their viability was reduced by 45% after 5 days (Figure 3C). Conversely, pertuzumab had no significant effect on shHER3 BxPC-3 cell growth (Figure 3C), demonstrating that HER3 knockdown *in vitro* induces resistance to pertuzumab therapy in pancreatic cancer cells.

To confirm the relationship between HER3 expression and pertuzumab therapeutic efficacy *in vivo,* mice were grafted with shHER3 or shCTRL BxPC-3 cells (Figure 3D-E). Pertuzumab significantly inhibited the growth of shCTRL BxPC-3 tumor xenografts and increased animal survival in comparison to untreated controls (p<0.0001 and p=0.0015) (Figure 3D). Conversely, only a slight inhibition of tumor growth (possibly due to activation of immune cells through ADCC or inhibition of HER2/HER1 heterodimers by pertuzumab) and no significant increase in survival were observed in mice xenografted with shHER3 BxPC-3 cancer cells and treated with pertuzumab in comparison to untreated controls (p=0.002; p=0.086) (Figure 3E). At the end of the experiment, HER3 expression was still down-regulated in shHER3 BxPC-3 tumor xenografts isolated from treated mice. These results indicate that BxPC-3 cell proliferation *in vivo* is partly HER3-dependent and that *HER3* knockdown *in vivo* abrogates pertuzumab therapeutic efficacy.

### Pertuzumab treatment induced an increase in HER3 expression

**Figure 4:**
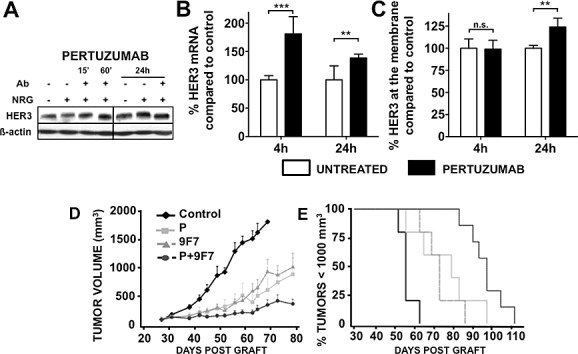
Effect of pertuzumab on HER3 protein (A) and mRNA (B) expression. BxPC-3 cells were pre-incubated with pertuzumab (50 μg/ml) and then with 100 ng/ml NRG1ß1 for 10 minutes. The total level of HER3 was then analyzed by western blotting (A). HER3 mRNA was quantified by q-PCR, normalized to a housekeeping gene and expressed relatively to neuregulin-treated cells (B. ** p < 0.01. The HER3 expression at the cell surface was analyzed by flow cytometry (C). Results are expressed as percentage of HER3 at the surface relative to HER3 at the surface of cells treated with neuregulin only. *** p < 0.001,** p < 0.01, n.s., not significant. The pertuzumab+9F7-F11 [anti-HER3 antibody] combination increases tumor growth inhibition in BxPC-3 pancreatic cancer cell xenografts (D-E). BxPC-3 cells were xenografted in nude mice that were then treated with pertuzumab, 9F7-F11 or both [10 mg/kg/each antibody; twice per week]. Results are presented as the mean tumor volume of each group (D) and as Kaplan-Meyer survival curves (E) (% of mice with a tumor volume < 1000 mm^3^ as a function of time). Bars = SEM.

HER3 expression after pertuzumab treatment was studied in NRG1β1-stimulated BxPC-3 cells (Figure 4). Pertuzumab did not affect HER3 protein level during the first hours of treatment but a slight increase was observed after 24h compared with untreated cells (Figure 4A). By qPCR analysis, an increase in HER3 mRNA was observed after 4 hours of pertuzumab treatment (Figure 4B), leading to an increase in HER3 expression at the cell surface after 24 hours (Figure 4C). These results demonstrate the ability of pertuzumab to increase HER3 expression.

### Combined treatment with pertuzumab and the anti-HER3 antibody 9F7-F11 enhances growth inhibition of pancreatic cancer xenografts

Based on the finding that pertuzumab increases HER3 expression, we compared the effect of the anti-HER3 mAb 9F7-F11 and of pertuzumab, which targets HER3 indirectly (Figure 4D), on BxPC-3 tumor xenograft growth. Both antibodies markedly slowed down tumor growth compared with the untreated group (p<0.001) and no significant difference was observed between antibodies (p=0.6488) (Figure 4D). The median survival was significantly delayed by 17 days in mice treated with 9F7-F11 and by 23 days in mice treated with pertuzumab in comparison to controls (Figure 4E).

Co-treatment with 9F7-F11 and pertuzumab inhibited tumor growth much more than each antibody alone (pertuzumab alone versus pertuzumab+9F7-F11 p=0.004). At the end of the 4-week treatment, tumor volume kept increasing in mice treated with pertuzumab or 9F7-F11 alone, whereas it remained quite stable in animals that received the pertuzumab+9F7-F11 combination. Median survival was longer in animals treated with the two-antibody combination than in control animals [gain of 41 days; p=0.0001] or in mice that received a single antibody (pertuzumab+9F7-F11 vs 9F7-F11 p=0.0013; pertuzumab+9F7-F11 vs pertuzumab p=0.0355).

### Pertuzumab and 9F7-F11 differentially affect HER2 and HER3 and downstream signaling pathways in pancreatic cancer cells

**Figure 5:**
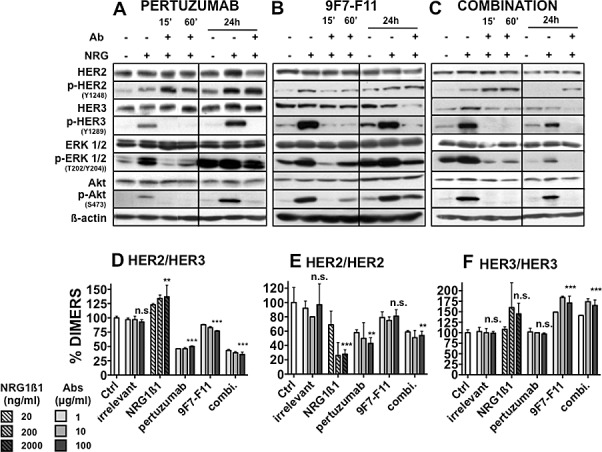
Effect of pertuzumab and of the anti-HER3 antibody 9F7-F11 on HER2, HER3 and downstream signaling pathways in BxPC-3 cells Cells were pre-incubated with pertuzumab [A], 9F7-F11 (B) or both antibodies (C) (50 μg/ml/each antibody for the indicated time) and then with 100 ng/ml NRG1ß1 for 10 minutes. The expression level of phosphorylated and total HER2, HER3, AKT and ERK was then analyzed by western blotting. Effect of pertuzumab and 9F7-F11 on (D) HER2/HER3, (E) HER2/HER2 and (F) HER3/HER3 dimer formation in NIH/3T3 HER2/HER3 cells. 10^5^ cells/well were incubated with increasing concentrations (1 to 100 μg/ml) of antibodies in serum-free medium for 30 minutes. The TR-FRET signal was expressed as Delta F665 (%) and then as dimer percentage (see “Materials and Methods”). Data are the mean ± SEM of 3 experiments performed in triplicate. P, ***<0.001, ** p < 0.01, n.s., not significant.

To assess the mechanisms underlying the role of HER3 in pancreatic cancer response to pertuzumab, HER2/HER3 expression, phosphorylation and downstream signaling were studied in NRG1β1-stimulated BxPC-3 cells after pertuzumab addition (Figure 5A). As expected, NRG1β1 stimulation induced phosphorylation of HER2 and HER3 and of the downstream signaling molecules ERK1/2 and AKT. Pertuzumab did not affect HER2 and HER3 expression during the first hour of treatment and only slightly after 24h of treatment in comparison to untreated cells. Conversely, it increased HER2 phosphorylation even after 24h of treatment and completely abrogated HER3, AKT and ERK1/2 phosphorylation. These results were confirmed *in vivo* in BxPC-3 tumor xenografts isolated from antibody-treated and control mice (Supplementary Figure. 1).

We then investigated the effect on BxPC-3 cells of direct HER3 targeting by 9F7-F11 and stimulation with NRG1β1 (Figure 5B). 9F7-F11 did not affect HER2 expression, but inhibited HER2 phosphorylation during the first 60 min of treatment. HER3 was significantly down-regulated after 24h of treatment and phosphorylation of HER3, AKT and ERK 1/2 was strongly reduced already after 15 min of treatment.

The pertuzumab+9F7-F11 combination had no effect on HER2 expression, but increased HER2 phosphorylation (Figure 5C), like with pertuzumab alone (Figure 5A). After 15 minutes of dual antibody treatment, as observed with 9F7-F11, HER3 was significantly down-regulated and HER3, AKT and ERK1/2 phosphorylation inhibited.

### Pertuzumab blocks HER2/HER3 hetero-dimerization and HER2 homo-dimerization without affecting HER3 homodimers

Using a recently described antibody-based TR-FRET assay [8], we analyzed the effects of pertuzumab and/or 9F7-F11 on HER2/HER3 dimerization in HER2/HER3 transfected fibroblasts. This cell line was chosen because it expresses at least ten-fold more HER2 and HER3 receptors than the pancreatic cancer cell lines and thus allows obtaining a better fluorescence signal to accurately investigate dimer formation. In comparison to untreated cells, incubation with pertuzumab led to a significant (50%) decrease of HER2/HER3 heterodimers (p<0.001)(Figure 5D). Incubation with the 9F7-F11 antibody also reduced the percentage of HER2/HER3 heterodimers, but to a lesser extent (25 %, p<0.001). Co-incubation with pertuzumab and 9F7-F11 was the most effective with a 64 % decrease in HER2/HER3 dimer concentration (p<0.001). Pertuzumab, but not 9F7-F11, decreased HER2 homodimers (Figure 5E). Conversely, 9F7-F11, but not pertuzumab, increased the percentage of HER3 homodimers (Figure 5F), possibly because 9F7-F11 could bring two receptor molecules in proximity. The antibody combination also increased HER3 homodimer concentration, an effect probably due to 9F7-F11. Incubation with NRG1β1 increased the percentage of HER2/HER3 heterodimers and HER3 “proximers” (at high doses), while it decreased the percentage of HER2 homodimers.

### Immunohistochemistry detection of HER2/HER3 co-expression in human PDAC

**Figure 6:**
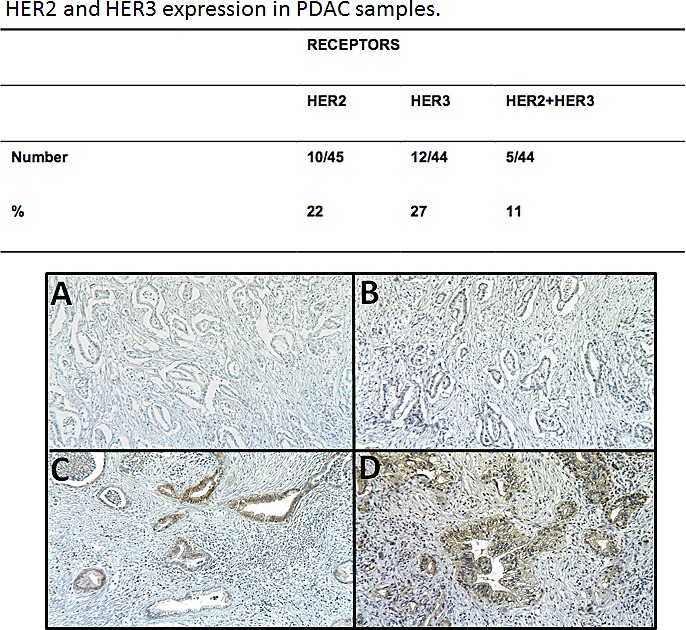
HER2 and HER3 expression was assessed by IHC in 42 pancreatic cancer samples A, HER2-negative case, B, HER3-negative case, C, HER2-positive case and D, HER3-positive case.

Finally, we investigated whether HER2 and HER3 are co-expressed in PDAC specimens from 45 patients by immunohistochemistry (IHC). HER2 was detected by IHC in 10/45 PDAC specimens (22%). HER2 expression was moderate in two of them and weak in the others (Figure 6). HER3 was detected in 12/44 PDAC (27%). In all cases, staining was weak but clearly positive. HER2/HER3 co-expression was observed in 5/44 PDAC (11%); specifically, 42% of the HER3-positive PDAC samples were also HER2-positive. HER2 and HER3 were mainly detected in the cytoplasm (Figure 6C and) and weak membrane expression was observed in several specimens.

## DISCUSSION

Our results demonstrate that HER3 is essential for pertuzumab efficacy in HER2^low^-expressing pancreatic tumor xenografts and suggest that the combination of this antibody with an anti-HER3 antibody could be a new potential treatment for pancreatic cancers that co-express HER2 and HER3. In our cohort of patients with PDAC, 11% of tumors co-expressed HER2 and HER3. However, the IHC method used for HER2 detection was optimized and validated only for breast and stomach cancer, thus it may not be sensitive enough to detect weak HER2 expression in PDAC. Consequently, we could have underestimated the number of patients with HER2-positive PDAC. Indeed, the pancreatic cancer cell lines BxPC-3, MiaPaCa-2 and Capan-1, which were classified as HER2-negative using the HercepTest (Dako) [6], showed moderate HER2 expression by using a more sensitive flow cytometry technique (Figure 1). Moreover, the finding that growth of mouse xenografts derived from such HER2^low^ cell lines was efficiently reduced by pertuzumab (Figure 3) suggests that pertuzumab could be effective in a wider population of patients with PDAC that have lower HER2 expression level than the one used to select eligible patients for trastuzumab therapy based on the Herceptest or Pathway HER2 assays. The development of a suitable method for evaluating HER2 expression in PDAC is a current project of our group.

Dimerization is essential for the activity of HER family members and plays a major role in driving the growth of several cancers [20]. As HER2 extracellular domain is always in the active conformation, HER2 is ready to interact with other ligand-activated HER partners. *In vitro* and *in vivo* experiments have demonstrated that HER2-containing heterodimers elicit greater mitogenic responses than HER2 homodimers. Thus, ligand-induced activation of EGFR or HER3 and formation of heterodimers with HER2 may play an important role in tumor growth and resistance to therapy. Inhibition of HER2/HER3 dimerization could represent a rational approach to anticancer therapy where HER3-mediated signaling plays a role in tumorigenesis. Pertuzumab is the first in a new class of drugs known as HER dimerization inhibitors and has already been tested in preclinical models of breast, prostate, lung, ovarian and colon cancers. Although most of these works reported that pertuzumab is efficient in HER2 over-expressing cancer cells, some found that it can inhibit tumor growth independently of HER2 protein expression level [21]. In a phase I clinical trial to assess the safety and pharmacokinetic profile of pertuzumab in patients with incurable, locally advanced, recurrent or metastatic solid malignancies, clinical efficacy was observed in two patients with advanced pancreatic and ovarian cancer that did not overexpress HER2 [22]. A phase II trial on pertuzumab in refractory ovarian cancers showed an increase in the median progression-free survival in patients with HER2-activated tumors compared to the group with HER2-inactivated cancers, independently of the HER2 overexpression status [23].

Our pre-clinical results corroborate these findings and suggest that HER dimerization inhibition might represent a novel clinical approach for pancreatic cancer treatment. Indeed, although HER2 expression was comparably low [approximately 15 000 receptors/cell] in the six pancreatic cancer cell lines used in this study, pertuzumab inhibited cell proliferation only in HER3-positive cells, probably through blockade of ligand-induced HER2/HER3 dimers. Pertuzumab efficacy in pancreatic cancer cells was greatly increased when exogenous NRG1 was added as stimulator, thus demonstrating that ligand-induced HER2/HER3 dimers strongly affect the fate of pancreatic tumor cells. The importance of HER2/HER3 dimers is strengthened by the high EGFR expression in the six cell lines. This finding indicates that, besides the role of EGFR/HER3 and HER2/EGFR dimers [13], also HER2/HER3 dimers are major drivers of tumorigenesis in HER2^low^ pancreatic cancer.

A short treatment (15 min) of BxPC-3 pancreatic cancer cells with pertuzumab was sufficient to abrogate HER3 and AKT phosphorylation, whereas no effect was observed on HER2 phosphorylation. This suggests that pertuzumab has an indirect effect on HER3, possibly through disruption of HER2/HER3 dimers and the consequent inhibition of HER3 activation and induction of HER3 stabilization at the cell surface. Treatment with pertuzumab (24 h) led to HER3 protein increase, which could be involved in bypassing the pertuzumab-induced HER2 inhibition, as observed by Garrett et al [24] in breast cancer cells treated with the HER2 inhibitor lapatinib, and by Nagumo et al [25] in ovarian cancer cells and breast cancer xenografts treated with pertuzumab.

HER3 seems to fine-tune the anti-tumor drug response [26] and thus could be used as a biomarker to select patients who might benefit from targeted therapy, as suggested for erlotinib in pancreatic cancer [27]. HER3-positive pancreatic cancers are more sensitive to erlotinib because it prevents transactivation of HER3 by EGFR and thus inhibits the PI3K/AKT signaling pathway, but not the ERK cascade, which is mainly mutated in pancreatic cancer. Similarly, the impact on HER3 expression in response to pertuzumab is driven through the PI3K pathway, but we suggest that HER2/HER3 heterodimers are mostly involved in this case. HER3 mRNA expression has been proposed also as a biomarker of pertuzumab response in ovarian cancer, but its role is unclear and controversial [10, 28, 29].

Finally, based on the results on HER3 activity following pertuzumab treatment, we compared the effects of direct (9F7-F11 mAb) and indirect (pertuzumab) targeting of HER3 in pancreatic tumor xenografts. Pertuzumab and 9F7-F11, had comparable effects on tumor growth, but the underlying cellular mechanisms were different. 9F7-F11 stimulated HER3 homodimer formation, probably due to the antibody bivalency, that could lead to unproductive HER3/HER3 association and subsequently to HER3 homodimer down-regulation and degradation. Conversely, pertuzumab treatment increased HER3 expression at the cell membrane and decreased the percentage of HER2/HER3 heterodimers. This finding provides a rationale for testing the use of HER3 inhibitors in combination with pertuzumab in PI3K/AKT-dependent pancreatic cancers. Indeed, co-treatment with pertuzumab and 9F7-F11 strongly increased their anti-tumor effect in the HER3-positive BxPC-3 xenograft model through enhanced HER3 down-regulation, disruption of heterodimers and stronger inhibition of the PI3K/AKT and ERK pathways. This suggests that a more complete and sustained inhibition of HER3 is necessary to block PI3K function and disable HER2 signaling to PI3K in HER2/HER3-expressing pancreatic cancer.

The therapeutic efficacy of pertuzumab in HER2-low pancreatic carcinomas needs now to be investigated in a Phase I clinical trial to be fully validated. Our findings provide evidence that HER3 expression in pancreatic cancer biopsies should be investigated as a biomarker of pertuzumab efficacy and to identify patients who might benefit from this therapy.

## MATERIALS AND METHODS

### Materials

Trastuzumab and pertuzumab were from Roche Pharma AG (Grenzach-Wyhlen, Germany). The anti-HER3 antibody 9F7-F11 was produced in our laboratory, as previously described [17]. The anti-HER2 mouse monoclonal antibody (mAb) FSP77 was kindly provided by N. Hynes [Basel, Switzerland]. Neuregulin 1 beta 1-extracellular domain (ECD) (NRG1β1) was purchased from RD Systems (Minneapolis, MN).

### Cell lines and culture

The BxPC-3, CFPAC-1, HPAC and MiaPaCa-2 cell lines were obtained from ATCC (Rockville, MD). The Capan-1APS cell line, which was derived from a xenograft of Capan-1 cells, was kindly provided by L. Buscail (Toulouse, France). The PancPec cell line was derived from a human pancreatic tumor specimen by Charles Theillet in our institute.

### Flow cytometry

The expression of EGFR, HER2 and HER3 was analyzed by flow cytometry using monoclonal antibodies against human EGFR [cetuximab], HER2 (FSP77) and HER3 (SC53279, Santa Cruz Biotechnology, Santa Cruz, CA) as previously described [6].

### Cell proliferation assay

The effect of NRG1β1 and pertuzumab on cell proliferation was evaluated using 3-[4,5-dimethylthiazol-2-yl]-5-[3-carboxymethoxyphenyl]-2-[4-sulfophenyl]-2H tetrazolium (MTS) and the electron coupling reagent phenazine methosulfate (PMS) as already described [17].

### Quantitative PCR for HER3 expression analysis

RNA was isolated using an RNeasy Mini kit (Qiagen) according to the manufacturer's protocol. RNA samples were first reverse transcribed using the M-MLV reverse transcriptase (Invitrogen) following the manufacturer's protocol. Gene expression was then assessed by quantitative PCR (qPCR) using a Light Cycler 480 SYBR Green I Master apparatus (Roche Applied Science). The primer sequences are presented in Supplementary data. PCR amplifications were performed in a standard 384-well plate format with a Roche LC480 real-time PCR detection system. For data analysis, the raw threshold cycle (CT) value was first compared to a standard curve to determine each cDNA relative concentration. These concentrations were then normalized to the housekeeping gene concentration and expressed relatively to the untreated samples.

### Short hairpin RNA constructs

Based on the work of Lee-Hoeflich et al. [8], two short hairpin oligonucleotides were chosen to knockdown HER3 mRNA levels (Supplementary Materials and Methods). The annealed shRNA sequences were digested with BamH I and EcoRI and inserted into the RNAi-Ready pSIREN-RetroQ Vector (Clontech). The control vector (shCTRL) pSIREN-shLuc was kindly provided by L. Le Cam and described previously [19]. pSIREN-shHER3 and pSIREN-shLuc, which contain the puromycin resistance gene, were transfected in the amphotropic packaging cell line AmphoPack-293 [Clontech]. Supernatants containing replication-defective virus particles were collected and used to infect BxPC3 cells. After 7 days of selection, cells were subcloned and selected based on the absence of endogenous HER3 protein expression.

### Western blot analysis

BxPC-3 cells were lysed and western blotting performed as previously described [18]. Membranes were incubated with the anti-human HER3 (Millipore, Billerica, MA) and anti-human HER2, ERK1/2, AKT, or anti-phosphorylated HER3, HER2, ERK1/2 or AKT antibodies (Cell Signaling Technology, Beverly, MA). Equal loading was assessed with an antibody against ß-actin (Cell Signaling Technology).

### HER2/HER3 dimer analysis

HER2/HER3 dimers were quantified using an antibody-based TR-FRET assay, as described [18], except for the detection of HER2 and HER3 homodimers (10 nM Trastuzumab-Lumi4™ Tb plus 10 nM Trastuzumab-d2 and 10 nM H4B-121-Lumi4™ Tb plus 10 nM H4B-121 d2, respectively).

### Tumor xenografts and treatment

All *in vivo* experiments were performed in compliance with the French regulations and ethical guidelines for experimental animal studies in an accredited establishment (Agreement No. C34-172-27). Six week/old female athymic mice, purchased from Harlan (Le Malcourlet, France), were injected subcutaneously into the right flank with parental shHER3 (3.5×10^6^) or control shLuc BxPC-3 cells (4.5×10^6^), Capan-1APS (10×10^6^), MiaPaCa-2 (5.3×10^6^), CFPAC-1 (5×10^6^), HPAC (5×10^6^) or PancPec (10×10^6^) cells. Tumor-bearing mice were randomized to different treatment groups (at least 6 animals/group) when tumors reached a volume of 100 mm^3^ and were then treated with 2 or 10 mg/kg pertuzumab, 10 mg/kg 9F7-F11 or the pertuzumab plus 9F7-F11 combination [10 mg/kg of each mAb]. Antibodies were given intraperitonally (i.p.) twice a week for 4 weeks. Tumor volumes were calculated by using the formula: D_1_ × D_2_ × D_3_ /2. For survival comparison, mice were sacrificed when tumors reached a volume of 1000 mm^3^.

### Immunohistochemistry

Surgically excised, formalin-fixed, paraffin embedded (FFPE) PDAC specimens were cut into 4-μm sections that were deparaffinized in xylene and hydrated in graded alcohols. For HER2, antigen retrieval was performed in CC1 buffer (Ventana) at 95°C for 20 min. Sections were then incubated with rabbit monoclonal anti-HER2 (clone 4B5, PATHWAY HER-2/neu, Ventana) for 20 min at 37°C. The antigen-antibody reaction was visualized by using UltraView DAB Reveal System and a Ventana Benchmark IHC staining automate. For HER3, antigen retrieval was performed at 97°C for 20 min in EnVision® Target Retrieval Solution High pH (Dako). Sections were then incubated at 37°C with mouse monoclonal anti-HER3 (clone DAK-H3-IC, Dako, Glostrup, Denmak) diluted at 1:50 for 2 h. The antigen-antibody reaction was revealed using EnVision® Flex DAB System and a Dako Autostainer Plus automate. IHC staining was interpreted by an expert pathologist who was blind to the patients’ information. HER2 and HER3 protein expression were semi-quantified using an arbitrary scale of increasing intensity (from 0 to 3).

### Statistical Analysis

The statistical analysis of tumor xenograft was performed using the STATA 11.0 software (StataCorp., College Station, TX) as described [17]. The statistical analyses of the TR-FRET and cell viability data were performed with the Prism GraphPad software (San Diego, CA) using one-way ANOVA followed by the Dunnett's test comparison with untreated cells).

## SUPPLEMENTAL MATERIAL, METHODS, TABLE AND FIGURES


