# Supplementary material for: HER3 as biomarker and therapeutic target in pancreatic cancer: new insights in pertuzumab therapy in preclinical models
Source: Oncotarget. 2014 Jul 17;5(16):7138–48. doi: 10.18632/oncotarget.2231 (PMC4196190; doi:10.18632/oncotarget.2231)
Supplement: Supplementary file 1 [file oncotarget-05-7138-s001.pdf]

HER3 as biomarker and therapeutic target in pancreatic cancer: new insights in pertuzumab therapy in preclinical models

Supporting Materials and methods

Quantitative-PCR HER3 mRNA determination

The sequences of the primer sets used for the analysis of HER3 mRNA are as follows: HER3, 5′-GTGGACTCGAGCAACATTGA -3′ (forward) and 5′-CCGTACTGTCCGGAAGACAT-3′ (reverse); HPRT was used as a housekeeping gene for normalization of HER3 gene expression. Primer sets for HPRT are as follows: F, 5′- GAC-ACT-GGC-AAA-ACA-ATG-CA -3′ (forward), 5′- CTT-CGT-GGG-GTC-CTT-TTC-ACC -3′ (reverse). An annealing temperature of 55 °C was used for all of the primers. PCRs were performed in a standard 384-well plate format with a Roche LC480 real-time PCR detection system.

Short hairpin RNA constructs

Two short hairpin oligonucleotides were chosen to knockdown HER3 mRNA levels at described in supporting materials and methods. 5'-GATCCCCAAGAGGATGTCAACGGTTATTCAAGAGATA ACCGTTGACATCCTCTTTTTTTTGGAAG -3' (forward; shRNA_HER3_1S) and 5'- AATTCTTCC AAAAAAAAGAGGATGTCAACGGTTATCTCTTGAATAACCGTTGACATCCTCTTGGG -3' (reverse; shRNA_HER3_1AS). The annealed shRNA sequences were digested with BamH I and EcoRI and inserted into the RNAi-Ready pSIREN-RetroQ Vector (Clontech).

Supplementary Table 1. Status of the six pancreatic cancer cell lines regarding the most common mutations in PDAC

	CFPAC-1	HPAC	BxPC-3	Capan-1	MiaPaCa-2	PancPec
KRas	M	M	WT	M	M	M
PI3KCA	WT	WT	WT	WT	WT	M
PTEN	-	-	-	-	-	-

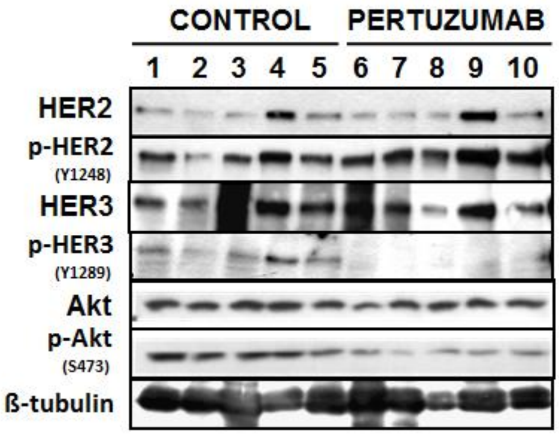

Figure 1: BxPC-3 cells were xenografted in nude mice that were then randomized in different groups (n=5/group) and treated twice with 10 mg/kg pertuzumab or sterile PBS (day 1 and 3 after the beginning of treatment). Tumors were resected at day 4. Cell lysates were analyzed by western blotting for total HER2, HER3 and AKT, phosphorylated HER2, HER3 and AKT, expression. Tubulin served as loading control.
